# Feedback on role model behaviour: effective for clinical trainers?

**DOI:** 10.1007/s40037-015-0184-x

**Published:** 2015-05-12

**Authors:** H.G.A. Ria Jochemsen-van der Leeuw, Margreet Wieringa-de Waard, Nynke van Dijk

**Affiliations:** Department of General Practice/Family Medicine, Academic Medical Center–University of Amsterdam, PO Box 22700, 1100 DE Amsterdam, The Netherlands

**Keywords:** Feedback, Role modelling, Post-graduate education, Faculty development, Educational assessment

## Abstract

**Aim:**

The aim of this study was to assess changes in role model behaviour of clinical trainers after giving personal feedback.

**Methods:**

First-year general practitioner (GP) trainees at two institutes for GP speciality training in the Netherlands were asked to complete an assessment of their clinical trainers: the Role Model Apperception Tool (RoMAT). The RoMAT consists of attributes of positive role modelling divided into two components (Caring Attitude and Effectiveness) and was scored on a 5-point Likert scale twice. After the first assessment moment, the trainers received their personal scores combined with the mean score of their peers. The trainers were divided into three performance groups: below average, average and above average.

**Results:**

Only the group with the lowest scores showed an improvement on the Effectiveness component of the RoMAT from 3.89 to 4.08 (*p* = 0.04) with an effect size of.52, showing a large effect. This pattern is confirmed by the number of trainers shifting from the below average performance group to the average (7) and above average (5) performance groups.

**Conclusion:**

Giving feedback to clinical trainers resulted in better scores on the Effectiveness characteristics. This indicates that role model behaviour of clinical trainers can be improved.

## Background

In clinical practice, trainees develop into competent physicians by working alongside clinical trainers. Besides being teachers, mentors or coaches, clinical trainers are also observed and imitated as role models when, for example, they are providing patient care or working together with other healthcare workers. To use clinical workplace learning as an educational tool to foster the development of trainees into competent professionals, it is therefore important to enhance the role model behaviour of clinical trainers [1].

To assist in this effort, we have developed and validated a tool to assess role model behaviour. This Role Model Apperception Tool (RoMAT) [2] consists of attributes of positive role modelling drawn from a systematic review of the literature [3]. Trainees can use the RoMAT to distinguish, through apperception, between positive and negative role modelling, and to assess clinical trainers’ role model performance. The RoMAT (See Appendix) consists of 17 items scored on a 5-point Likert scale and divided over two components: ‘Caring Attitude’ and ‘Effectiveness’. ‘Caring Attitude’ clusters items that reflect characteristics of the relationship between trainers and their patients, trainees and others. ‘Effectiveness’ represents items relating to the ability of trainers to provide their patients and trainees with what they need. Both components include an equal number of items addressing personal, teaching and clinical qualities, with high reliabilities [2].

Maker and colleagues [4] showed a positive effect of personal feedback on role model behaviour of faculty members of a surgical department. Van Es and colleagues [5] described recommendations for improving acceptance of feedback, for instance by providing trainers with the score ranges of their colleagues, especially if combined with narrative comments.

The aim of this study was to replicate the results of Maker and colleagues [6] while taking into account the recommendations of Van Es et al., by assessing changes in role model behaviour of GP trainers, using the RoMAT to give personal feedback on the role model function.

## Methods

In the Netherlands, trainees spend 3–4 days a week during their first and third years of GP speciality training working at GP practices under the supervision of their GP trainers. Throughout their training, trainees spend one day a week at one of the eight institutes for GP speciality training, where they are instructed by teachers. Each year, GP trainers attend eight training days at these institutes.

We invited first-year GP trainees, starting between September 2012 and March 2013 at two institutes—the Academic Medical Center of the University of Amsterdam (AMC-UvA) (*n* = 91) and the VU University Medical Center Amsterdam (VUMC) (*n* = 79)—to participate in our study. We asked the respondents to complete the RoMAT for their trainers at 6 months (T_1_) and 12 months (T_2_) after the start of their first training year as part of the bi-annual evaluation of the trainers. Each trainee rated one trainer. In case of training practice with several trainers, trainees were asked to rate their main trainer. All respondents were informed that this newly introduced tool would be part of a study, that participation in the study was voluntary and that questionnaires would be coded in order to prevent responses being traceable to individual respondents. Ethical approval was sought and granted by the Dutch Association for Medical Education (NVMO file number 226).

After the first assessment, we calculated means and SD scores for the overall scales and of each item separately for all trainers at the same institute. As written feedback, the trainers received their personal scores combined with the mean score of their peers. Also, trainees were encouraged to discuss the scores they noted with their trainers.

We divided the trainers into three performance groups for each of the components (i.e. Caring Attitude and Effectiveness) based on their T_1_ scores: below average performance (≥ 1 SD below the mean), average performance (− 1 SD <mean <+ 1 SD) and above average performance (≥ 1 SD above the mean). For each group we compared the scores of both components separately at T_1_ and T_2_ with a paired t-test and calculated the effect sizes (ES) by dividing the mean difference by the common SD. A p-value < 0.05 was considered a statistically significant change; an ES of < 0.3 was regarded as small, between 0.3 and 0.5 as moderate, and > 0.5 as large. We also calculated the number of trainers in each group (below, mean and above average) before and after feedback.

## Results

A total of 76 trainees responded at both T_1_ and T_2_, namely 68 (67 %) trainees at the AMC–UvA and 8 (11 %) at the VUMC (Table 1), resulting in 76 available scores of trainers. The average score of all participants on the Caring Attitude component at T_1_ was 4.63 (SD 0.36) and at T_2_ 4.62 (SD 0.37). On the Effectiveness component the score at T_1_ was 4.40 (SD 0.38) and at T_2_ 4.41 (SD 0.42). No significant change in scores on the Caring Attitude component was established for the three groups. The scores on the Effectiveness component in the below average performance group increased from 3.89 to 4.08 (*p* = 0.04) with an ES of.52, showing a large effect. This pattern was confirmed by the number of trainers shifting from the below average performance group to the average (7) and above average (5) performance groups.

**Table 1:** Results for the three groups^a^ before (T_1_) and after (T_2_) feedback for both components of the Role Model Apperception Tool (RoMAT)

Component of RoMAT		Group 1 (≥ 1 SD below the mean)	Group 2 (− 1 SD <mean < 1 SD)	Group 3 ( ≥ 1 SD above the mean)
Caring Attitude	Mean score (SD) T1	4.01 (0.14)	4.71 (0.16)	5.00 (0.00)
	Mean score (SD) T2	4.15 (0.32)	4.71 (0.25)	4.82 (0.38)
	P	0.134	0.932	0.088
	ES^b^	0.41	0.01	0.47
	Number of trainers at T1/T2	15/13	46/51	15/12
Effectiveness	Mean score (SD) T1	3.89 (0.11)	4.46 (0.21)	4.96 (0.07)
	Mean score (SD) T2	4.08 (0.37)	4.45 (0.38)	4.75 (0.36)
	P	**0.04** ^c^	0.92	0.10
	ES^b^	**0.52**	0.02	0.55
	Number of trainers at T1/T2	18/11	47/49	11/16

^a^The classification of the trainers in three performance groups for both components (Caring Attitude and Effectiveness): (1) Below average performance (≥ 1 SD below the mean), (2) Average performance (− 1 SD < mean <+ 1 SD) and (3) Above average performance (≥ 1 SD above the mean)

^b^Effect Size (ES) = Mean_difference_/SD_difference_ [8]

^c^Significant difference at *p* < 0.05

## Discussion

After personal feedback, only the group of GP trainers with the lowest scores showed an improvement on the Effectiveness component of the RoMAT. This pattern is confirmed by the number of trainers shifting from the group with below average performance to the average and above average performance groups. The improvement on the Caring Attitude component was not statistically significant, maybe due to the occurrence of a ceiling effect. Trainers received high scores on the Caring Attitude at the start of a traineeship, probably because GP trainers, in contrast to many clinical specialists, voluntarily choose to become a trainer and are thus very motivated to welcome trainees. Attributes of the Caring Attitude component represent this behaviour. In contrast, the Effectiveness scores in comparison to the Caring Attitude scores at T_1_ start low and increase; in the below average performance group, this increase is significant over the second 6-month period, showing a large effect at T_2_. In a previous study, trainees just starting their traineeships also rated their clinical trainers with higher scores on the Caring Attitude component than on the Effectiveness component, suggesting that, when starting their traineeship, they are most sensitive to the aspects of the Caring Attitude component and have not yet discovered possible negative characteristics of their trainers. Trainees seem to focus more on attributes of the Effectiveness component as they progress through their traineeship, indicating that trainers with higher scores on the Effectiveness component are better role models for preparing trainees to become independent GPs [6]. In other words, the aspects valued by trainees seem to shift over time, with aspects related to a safe learning environment being more important in the beginning and aspects related to becoming an independent GP being more important in a later phase. Nevertheless, in the groups with above average performance, the scores were already high on both components at the start, resulting in regression to the mean at T_2_. Although all scores show the same pattern and regression to the mean is to be expected to influence all results, only the improvement of the Effectiveness score in group 1, the low performance group, showed a significant rise with a large effect, confirmed by the largest shift of trainers from one group to the other.

The results of our study are consistent with those of Maker and colleagues with regard to the improvement in trainers with the lowest scores and in the number of trainers shifting from the lowest to the highest scores [4]. However, there are two important differences. Firstly, the surgical trainers also improved their overall score. Secondly, the surgical trainers showed improvement on four attributes that are similar to attributes of both the Caring Attitude and the Effectiveness component, while GP trainers only improved on the characteristics of the Effectiveness component. These differences might originate from contrasting conditions for GP and surgical trainers. GP trainers are volunteers and have to attend eight training days each year, so they are highly motivated and already focussed on being trainers. Furthermore, GP trainees have only one or two GP trainers as their role models at any one time, making it difficult to compare and to distinguish between positive and negative role modelling, while they also have to discuss their feedback with their trainers. This possibly results in high scores at the start of their training. Surgical trainees, working alongside surgeons who at the same time function as their trainers, have more opportunities to make comparisons and their assessments are anonymous. They therefore tend to give their trainers lower scores, resulting in more opportunities for an increase in scores.

A more recent study by Maker and colleagues showed that trainers with the lowest evaluations even further improve towards being a role model by way of a detailed feedback letter summarizing trainer performance, compared with role models (trainers with the highest performance scores) [7].

## Limitations

There was a low response in completing the RoMAT for the second time at one of the institutes, probably because the form was not sent off together with the standard evaluation, but in a separate email with only one reminder and because participation was voluntary. Bias can be expected as a result of the low response; trainees may not have responded in fear of the consequences for their evaluation by the trainer at the end of their training year. It may be that trainees who did respond were more positive about their trainers, which may have restricted the effect.

Further research with higher response rates is needed. To attain higher response rates, it might be helpful to implement the RoMAT, also in clinical settings, and to monitor both trainees’ and trainers’ results.

## Implications for future education

This study indicates that the RoMAT can be used to evaluate the effectiveness of educational interventions that are designed to improve role model behaviour of trainers.

## Conclusion

Giving feedback to trainers resulted in better scores on the Effectiveness component of the RoMAT for trainers from the below average performance group. This outcome seems to indicate that trainees are able to use the RoMAT to distinguish between positive and negative role modelling, and that the role model behaviour of GP trainers can be improved.

### Funding/Support

This study was funded by CASH (Committee for Activities to promote the Education of General Practitioners).

## Appendix. The Role Model Apperception Tool (= RoMAT) [3]

**Table:**

	CA/EF^a^	My Clinical Trainer…	Strongly disagree	Disagree	Neutral	Agree	Strongly agree
1	EF	Has excellent clinical reasoning skills	1	2	3	4	5
2	CA	Conveys empathy for patients	1	2	3	4	5
3	CA	Communicates well with patients and relatives	1	2	3	4	5
4	EF	Understands learners’ needs and is committed to the growth of learners	1	2	3	4	5
5	CA	Establishes rapport with learners	1	2	3	4	5
6	CA	Has a positive attitude towards learners	1	2	3	4	5
7	CA	Demonstrates enthusiasm for one’s work	1	2	3	4	5
8	CA	Is patient	1	2	3	4	5
9	CA	Has a positive interaction with other healthcare workers	1	2	3	4	5
10	EF	Makes learning exciting and stimulating	1	2	3	4	5
11	EF	Has self-confidence	1	2	3	4	5
12	CA	Is available to learners	1	2	3	4	5
13	CA	Is honest and has integrity	1	2	3	4	5
14	EF	Has leadership qualities	1	2	3	4	5
15	EF	Is aware of his/her role model status	1	2	3	4	5
16	CA	Is nice and easy to work with	1	2	3	4	5
17	EF	Is professionally competent in difficult clinical situations and able to cope with adversity	1	2	3	4	5

^a^Components of the RoMAT: Caring Attitude (*CA*) and Effectiveness (*EF*)
